# Left ventricular cardiac myxoma and sudden death in a dog

**DOI:** 10.1186/s13028-016-0222-7

**Published:** 2016-06-22

**Authors:** Maria Irene de Nijs, Aryan Vink, Wilhelmina Bergmann, Viktor Szatmári

**Affiliations:** 1 Veterinary Practice Schouwen-Duiveland, Grevelingenstraat 17, 4301 XZ Zierikzee, The Netherlands; 2 Department of Pathology, University Medical Centre Utrecht, Heidelberglaan 100, Utrecht, 3584 CX The Netherlands; 3 Department of Pathobiology, Faculty of Veterinary Medicine, Utrecht University, Yalelaan 1, Utrecht, 3584 CL The Netherlands; 4 Department of Clinical Sciences of Companion Animals, Faculty of Veterinary Medicine, Utrecht University, Yalelaan 108, Utrecht, 3584 CM The Netherlands

**Keywords:** Aorta, Heart, Neoplasia, Obstruction

## Abstract

**Background:**

Myxoma is a very rare benign cardiac tumor in dogs. This is the first description of a cardiac myxoma originating from the left ventricular outflow tract, presumably causing sudden death.

**Case presentation:**

A previously healthy 12-year-old male West Highland white terrier was found dead during its 1-week stay in a kennel. The dog was known to have a cardiac murmur. On necropsy, a pedunculated neoplasia was found attached to the interventricular aspect of the left ventricular outflow tract, resulting in almost complete obstruction of the aorta. As this was the only abnormality identified, the tumor was considered as the cause of sudden death. Histopathologic findings were compatible with a myxoma.

**Conclusions:**

Benign intraluminal tumors of the heart are very rare in dogs, but may have fatal consequences. Echocardiography could have revealed the cause of the cardiac murmur of this previously asymptomatic dog. Surgical removal could have been possible, as the tumor was pedunculated.

## Background

According to the definition of the World Health Organization myxoma is a neoplasm composed of stellate to plump bland mesenchymal cells set in a myxoid stroma [1]. As myxoid cells show cellular features of mesenchymal cells, the origin of the tumour is most likely the endocardium, but the exact histogenesis of myxomas is still unclear [1]. Cardiac myxoma is a rare, benign tumor in the dog [2–9]. The first report of a canine cardiac myxoma was published in 1959 [8], subsequently followed by a few case reports of myxomas involving the right atrio–ventricular valve [2, 4, 5, 7]. Myxoma in the left ventricle is even rarer, and has only been described once when it caused an obstruction of the left ventricular inflow tract of a canine heart [6]. In humans left ventricular myxoma is also a rarity, despite the fact that myxoma is the most common type of cardiac tumor in people [1, 10–15]. Fatal outcome has also been reported in humans [10].

## Case presentation

**Fig. 1:**
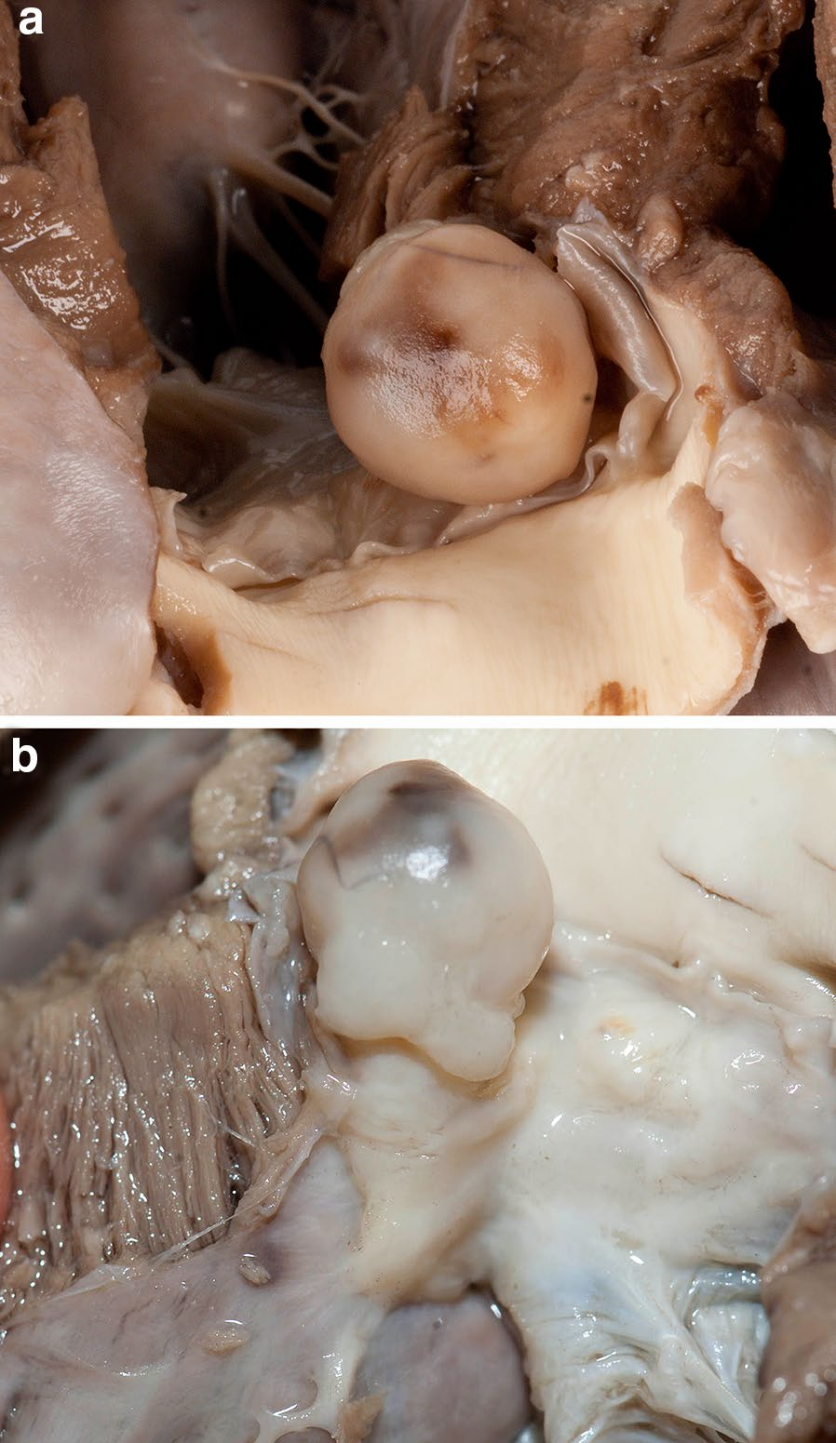
Left ventricular outflow tract showing an intracardiac mass. Formalin-fixed specimen. **a** The opened ascending aorta is obstructed by the myxoma (view from the aorta towards the left ventricular outflow tract). The aorta is almost completely obstructed by a round, smooth-surfaced mass. The mass is touching the aortic valves. **b** The origin of the pedunculated mass can be seen arising from the interventricular septum at the base of the right coronary leaflet of the aortic valve and protruding through the valve (view from the left ventricular free wall towards the interventricular septum)

A 12-year-old male West Highland white terrier was found dead after a week stay in a kennel. According to the owner and the animal caretakers the dog was normal prior to its sudden death. When the dog was last examined by a veterinarian, a few months prior to the sudden death, a systolic cardiac murmur was detected. No further diagnostic tests were performed at that time. The owner brought the cadaver to another veterinarian (MdN) to request necropsy. On post mortem examination, a 1 × 1 × 1 cm white firm to elastic smooth-surfaced pedunculated mass was found arising from the interventricular septum and protruding into and obstructing the aorta (Fig. 1). The cardiac mass caused an almost complete obstruction of the left ventricular outflow tract at the level of the aortic valves (Fig. 1). No other major abnormalities were detected on gross examination of the dog. The heart and lungs were removed and submitted for pathological examination.

**Fig. 2:**
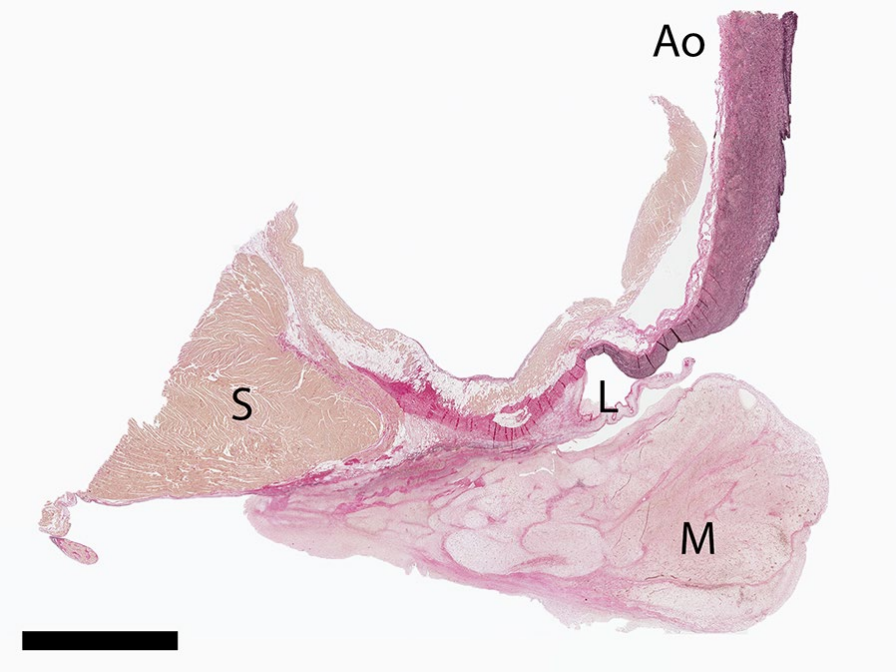
Photomicrograph of the myxoma in the left ventricular outflow tract. An overview showing that the myxoma (*M*) is attached to the endocardium just proximal of the aortic valve leaflet (*L*). The interventricular septum (*S*) and the proximal part of the aorta (*Ao*) are indicated. Elastin-van Gieson stain. *Bar* 5 mm

**Fig. 3:**
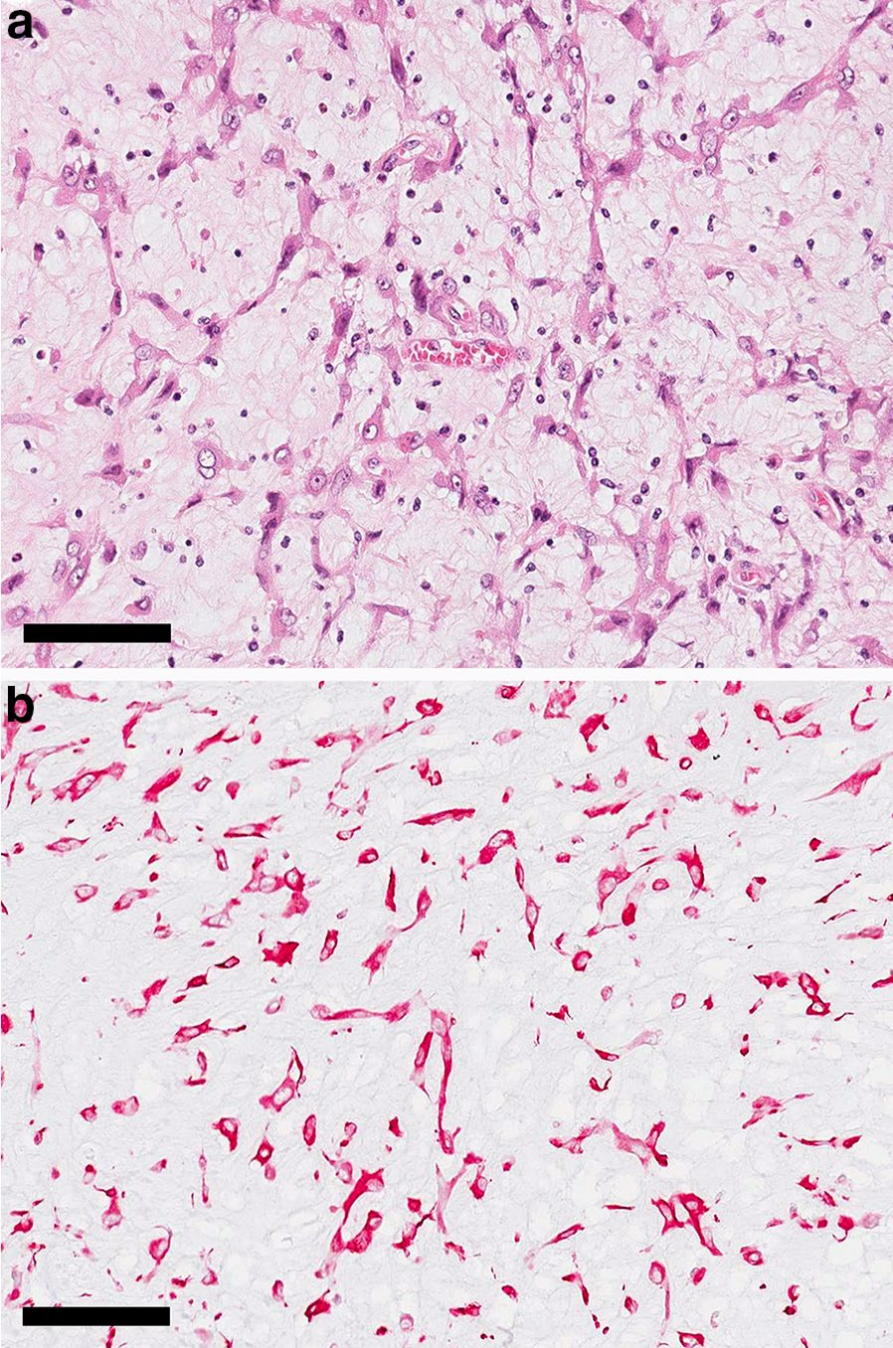
Photomicrograph of the myxoma. **a** The myxoma is histologically composed of spindle- and stellate-shaped cells embedded in a myxoid intercellular matrix. Some syncytia are evident. Hematoxylin & eosin stain. *Bar* 100 µm. **b** The myxoma cells stain positive (*red*) by vimentin immunohistochemistry. *Bar* 100 µm

On gross pathology, the intracardiac mass was attached to the endocardium just proximal to the aortic valve leaflet (Fig. 2). Histologically the mass consisted of spindle to stellate cells with abundant eosinophilic cytoplasm, so called “myxoma cells”, which formed bundles in a myxoid intercellular matrix (Fig. 3). The cells were 15–25 µm in diameter, with a centrally placed, oval, vesicular nucleus and a moderately sized nucleolus. The myxoma cells occasionally formed a syncytium; the diagnostic feature of a cardiac myxoma [1, 11]. There was a moderate anisocytosis and anisokaryosis present and there was one mitotic figure found in 10 high power fields (400×). Hemosiderin-laden macrophages were present around some capillaries. Extravasation of neutrophilic granulocytes was observed in the area beneath the endocardium. The infiltration of the superficial regions of the mass with neutrophil granulocytes was probably caused by the mechanical irritation (shear stress) as the result of a presumably severely increased blood flow velocity in the stenotic left ventricular outflow tract.

Immunostaining was performed to further characterize the neoplasia. The myxoma cells expressed vimentin and were negative for smooth muscle actin (Fig. 3). For the vimentin immunostaining the slides were pretreated by boiling citrate buffer (pH 6.0) for 20 min, for the α smooth muscle actin stain no pretreatment was used. Slides were incubated with an anti α smooth muscle actin (Sigma, clone 1A4, 1:1600) or an anti-vimentin (Dako, clone V9, 1:400) monoclonal antibody for 1 h at room temperature (i.e. around 20 °C) followed by incubation with Brightvision poly AP-anti-mouse IgG (Immunologic) for 30 min at room temperature. The signals were visualized with Liquid Permanent Red Chromogen (Dako).

Histopathologic examination of lung tissue revealed severe hyperemia, probably as a result of acute circulatory failure.

## Conclusions

Cardiac neoplasia is rare in dogs with a reported incidence of 0.1–0.2 % [3, 9, 16]. The vast majority of cardiac tumors are primary (84 %) and only a small proportion is the result of metastasis [9]. The most common primary cardiac neoplasia is hemangiosarcoma, followed by aortic body tumor (chemodectoma) [3, 9]. Other types of cardiac tumors, such as malignant lymphoma and mesothelioma, are very rare in the dog [2–9, 17–21]. Most often, cardiac tumors lead to pericardial effusion and subsequently to cardiac tamponade, which may result in ascites through splanchnic congestion, or to exercise intolerance, syncope or shock through low cardiac output [3, 9]. Less commonly, mass effect of the tumor leads to syncope or to pulmonary or splanchnic congestion by obstructing blood flow either due to intraluminal tumor growth or external compression of cardiac structures or great vessels [2–9, 17–21]. Infiltrative tumor growth may result in systolic dysfunction of a ventricle [9]. Arrhythmias are another possible consequences of cardiac neoplasia [9]. None of the above mentioned clinical signs were observed in the dog of the present case report.

There are only a few case reports in the veterinary literature describing canine cardiac myxomas. The clinical signs depend on the anatomical location and size of the mass. Dyspnea as a result of cardiogenic pulmonary edema was caused by a myxoma in a 4-year-old dog; the neoplasia involved the mitral valve apparatus so consequently led to a severe stenosis of the mitral valve [6]. In dogs where the mass involved the tricuspid valve, ascites developed as a result of congestive right sided heart failure [2, 7]. In another dog, whose mitral valve was affected by a myxoma, syncope was the presenting sign, and echocardiography revealed a dynamic left ventricular outflow tract obstruction and mitral valve regurgitation [22]. Similarly, exertional syncope was the presenting sign in a different dog, where the myxoma was located in the right ventricular outflow tract, causing its obstruction [4]. None of the previously reported clinical signs of myxoma was noticed in the dog of the present case report.

According to the authors’ knowledge, a left ventricular myxoma causing an outflow tract obstruction or a myxoma causing sudden death in a dog has not been reported previously. Whether the direct cause of death was a mechanical obstruction of blood flow in the aorta or a ventricular tachyarrhythmia secondary hypothesised myocardial hypoxia, remains unknown. Myocardial hypoxia might have been present as a consequence of obstruction of a coronary artery by the mass. In a similar case to our own, sudden death was described in a dog with a myxosarcoma, which caused left ventricular outflow tract obstruction, however, in this case the tumor was malignant [21].

Ante mortem diagnosis of this cardiac neoplasia would have been possible with echocardiography [4, 6, 7, 9, 12, 15]. Surgical resection of the neoplasia could have been subsequently attempted to prolong the dog’s life. Surgical removal of a mass from the left ventricle in a dog has not been reported but surgical resection has been described using open heart surgery with cardio-pulmonary bypass at the anatomical location where the fibrous ridge of subvalvular aortic stenosis is localized, i.e. at the same location where the mass in the present dog had developed [23]. Successful surgical removal of cardiac myxomas of right atrial and right ventricular origins has been described in two cases. One of which the right atrial myxoma was removed, but the dog lived only 36 h thereafter [7]. In a second dog, a myxoma from the right ventricular outflow tract was resected, and the dog lived another 2 years with a good quality of life [4].

The current case study suggests that the cause of a heart murmur in elderly small breed dogs, cannot be assumed to be degenerative valvular disease. Echocardiography might be helpful as further investigation to reach an ante-mortem diagnosis in dogs with a murmur, particularly because potentially treatable diseases may be found.
